# First-Principles Research on Hard Segment Regulation of Field-Induced Charge Transfer in Polyaniline-Grafted Polyurethane Copolymers

**DOI:** 10.3390/molecules31203751

**Published:** 2026-10-19

**Authors:** Qi Wang, Zhiyi Pang, Yi Li, Ming Yang, Shangshi Huang

**Affiliations:** 1 University Engineering Research Center of Advanced Technologies and Materials Applicated in Transportation, College of Traffic and Transportation, Nanning University, Nanning 530200, China; 2 State Key Laboratory of Power System, Department of Electrical Engineering, Tsinghua University, Beijing 100084, China; 3 Faculty of Intelligent Manufacturing, Nanning University, Nanning 530200, China; 4 School of Electrical Engineering, Guangxi University, Nanning 530004, China

**Keywords:** polyaniline-grafted polyurethane copolymer, density functional theory (DFT), field-induced charge transfer, hard segment, conductive coating

## Abstract

Lightning strikes pose an increasing threat to wind turbine blades, whose insulating GFRP laminates and polyurethane coatings accumulate surface charge with no dissipation pathway. Polyaniline-grafted polyurethane (PANI-PU) copolymers are being explored as conductive coating materials that dissipate charge under high electric fields. This research used density functional theory to compare three graft copolymers differing only in the diisocyanate hard segment, HDI, HMDI and IPDI, under a static field up to 205.7 kV/mm along the backbone with all other conditions identical. The response was analysed by Bader charge partitioning, differential charge density, orbital energetics and surface potential. HMDI-PANI transfers the largest net charge, 0.75 e, keeps its PU hydrogens near neutral at 0.10 e, spreads charge most diffusely with an index of 122.6, and shows the largest gap narrowing, 0.92 eV. HDI-PANI polarizes twelve free C-H bonds, raises its polarizability by 4.0 a.u. while its dipole falls by 0.25 D, and narrows its gap from 2.19 to 1.66 eV. IPDI-PANI reorients its methyl groups, giving the largest polarizability increase, 9.0 a.u., and dipole enhancement, 1.29 D, without a matching gain in transfer, 0.68 e. These results identify the dicyclohexylmethane hard segment as the architecture maximizing transfer efficiency, providing a molecular-level design basis for conductive graft copolymer coatings.

## 1. Introduction

Wind turbine blades are the components most frequently struck by lightning during operation. A single blade can experience multiple lightning attachments over its service life, with strike currents reaching tens to hundreds of kiloamperes [1,2,3]. Current protection relies on lightning receptors and down conductors embedded at discrete points along the blade [4,5,6]. These point-type protectors intercept the lightning leader but cannot cover the full blade area. The glass-fiber-reinforced polymer (GFRP) skin between the receptors remains electrically exposed. As turbine capacities increase and blade lengths exceed 100 m, the unprotected area grows, and the probability of lightning attachment to unprotected regions rises sharply [7,8].

The structural design of the blades compounds the lightning problem. GFRP laminates and the epoxy matrices that bond them are electrical insulators under normal operating conditions [9]. When lightning strikes an unprotected region, the arc root temperature exceeds 20,000 K in microseconds. The polymer matrix pyrolyzes, internal pressure builds from vaporized moisture and decomposition gases, and delamination propagates along the laminate interfaces [10,11]. Because the blade cannot conduct the lightning current away, the entire discharge energy is deposited locally. The damage is rarely a single event. Partial-discharge tracking along internal voids and manufacturing defects accumulates over successive strikes and eventually forms conductive carbonized paths that make the blade more vulnerable to the next event [12]. Post-strike inspection data show that over 60 percent of blade lightning damage involves delamination or debonding rather than direct puncture, confirming that structural failure, not just surface erosion, is the dominant degradation mode [13].

The blade surface is the first material layer that interacts with the lightning environment, so its electrical behavior determines the outcome of a strike. Polyurethane (PU) coatings are the industry-standard surface finish for blades, applied to resist rain erosion, block UV degradation, and maintain aerodynamic smoothness [14]. These coatings are electrical insulators with volume resistivities typically above 10^12^ Ω**·**cm [15]. Under high-voltage stress from thundercloud fields or nearby lightning leaders, charge accumulates on the coating surface through corona and brush discharge mechanisms [16]. The accumulated surface charge distorts the local electric field, lowers the effective breakdown threshold of the underlying GFRP, and can initiate surface flashover that guides the lightning channel toward the blade [17,18]. A coating designed for mechanical protection becomes an electrical liability. Instead of mitigating the threat, the insulating PU film turns the blade surface into a charge trap and, in some field configurations, a preferential attachment point.

The search for conductive or semi-conductive coatings that can dissipate surface charge has produced considerable experimental work [19,20]. Carbon-based fillers such as carbon black, carbon nanotubes, and graphene have been dispersed in PU matrices to reduce resistivity [21,22,23]. Filler-based approaches, however, face a trade-off. Achieving sufficient conductivity typically requires loadings high enough to compromise the mechanical flexibility and erosion resistance that make PU the coating of choice. An alternative strategy grafts intrinsically conductive polymers directly onto the PU backbone. Polyaniline (PANI), in particular, has been incorporated into PU copolymers to create conductive pathways while preserving the mechanical properties of the matrix [24,25,26]. But most of this research evaluates performance through bulk conductivity measurements and mechanical testing [27,28]. These techniques cannot resolve the electronic structure at the graft interface, the spatial distribution of field-induced charge, or how different hard segment architectures regulate electron transfer. DFT calculations access these properties directly. The wavefunction, the charge density, and the orbital energetics control charge transport. Closing the gap between macroscopic measurement and molecular-level electronic structure would move the design of conductive coatings from trial-and-error formulation toward mechanism-driven engineering.

In this study we compared three PANI-grafted polyurethane copolymers that differ only in their diisocyanate hard segment. DFT calculations and wavefunction analysis were employed to characterize the electronic structure and the field-induced charge redistribution in each system. The comparison of chain flexibility and substituent polarity across the three architectures revealed whether charge transfer follows a diffuse-uniform pattern or a localized-oscillatory one. Specifically, we investigated the electronic response of the graft interface and the spatial charge redistribution across the copolymer under an external electric field. Based on the results, we identified three distinct charge regulation mechanisms and the structure–property relationships that control the spatial distribution of transferred charge. This study offers a design principle for conductive PU coatings and provides a theoretical basis for selecting hard segment architectures in field-responsive graft copolymers.

## 2. Results

### 2.1. Interfacial Bond Strain and Molecular Polarization Under External Electric Field

**Figure 1:**
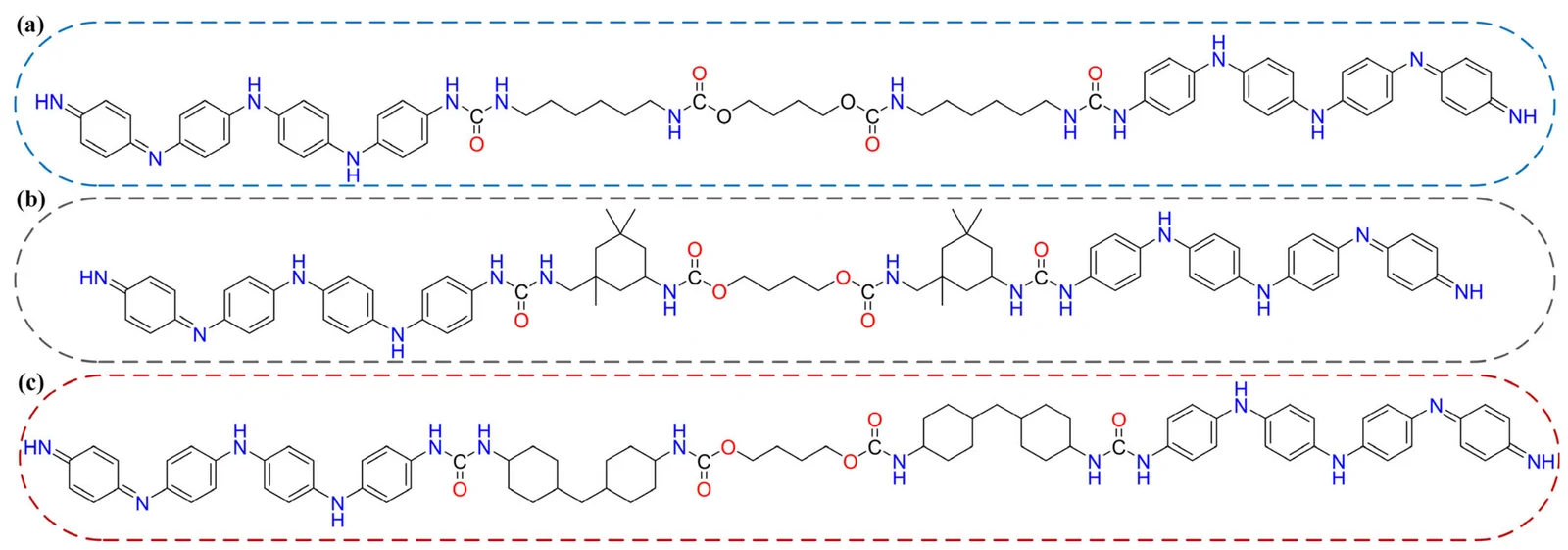
Molecular structures of the three polyaniline-grafted polyurethane copolymers with HDI, IPDI, and HMDI hard segments in (**a**–**c**).

This research provides two equivalent graft junctions, R_1_ and R_2_, for probing differential bond responses under an oriented external field, and establishes a continuous donor–acceptor pathway through the hard–soft–hard backbone, in which the urethane carbonyls and ether oxygens act as donors and the PANI π-systems as acceptors. Because the PANI termini and the central PTMG block are identical across the three systems, any variation in charge transfer must originate solely from the diisocyanate hard segment, enabling an unambiguous structure–property correlation. The terminal PANI chains also act as natural caps that avoid boundary artifacts such as dangling bonds or hydrogen terminations. The molecular structures of the three copolymers are illustrated in Figure 1, where (a) HDI-PANI features a flexible linear hexamethylene chain, (b) IPDI-PANI contains a cycloaliphatic ring with two pendant methyl substituents, and (c) HMDI-PANI adopts a symmetric dicyclohexylmethane scaffold.

**Figure 2:**
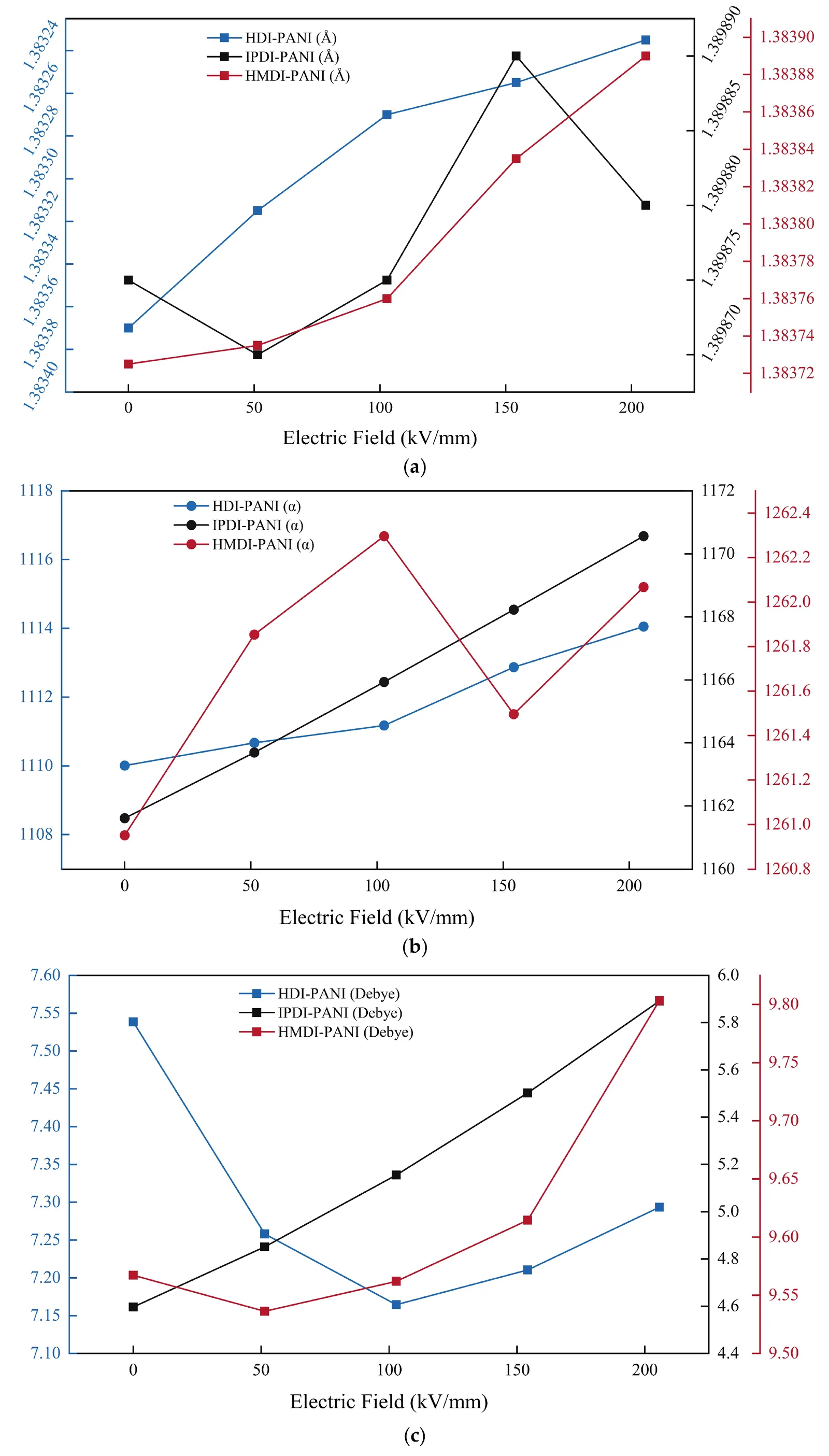
Field dependence of the averaged junction bond length R_a_, the polarizability and the dipole moment of HDI-PANI, HMDI-PANI and IPDI-PANI, under a static field from 0 to 205.7 kV/mm along +X, in panels (**a**–**c**). R_a_ is the mean of the two C–N bonds R_1_ and R_2_. Units are angstroms, atomic units and Debye. (**a**) Averaged graft-junction bond length R_a_ against the applied field, in angstroms; (**b**) Molecular polarizability in atomic units over the same field range; (**c**) Dipole moment in Debye as a function of the field.

Figure 2a shows the field dependence of the averaged graft-junction C–N bond length R_a_, defined as the mean of R_1_ and R_2_, where R_1_ and R_2_ are the two C–N bonds connecting the isocyanate carbon of the diisocyanate hard segment to the terminal NH nitrogen of each PANI chain. Table 1 lists the corresponding R_1_ and R_2_ values over the applied field range of 0 to 205.7 kV/mm along the +X molecular axis.

The C-N bonds that connect PANI to the PU backbone sit at the exact point where charge crosses from donor to acceptor. Their length is a direct structural reporter of local electron density. A shorter bond means higher electron density in the bonding region and greater bond order. A longer bond means electron depletion.

The three systems show different responses. HDI-PANI produces the smallest overall modulation. R_1_ lengthens by 0.00009 Å, while R_2_ shortens by 0.00036 Å. The signs are opposite because the +X field drives charge from left to right, so density arriving at R_2_ from the PU donor side compresses that bond while density leaving R_1_ stretches it. The hexamethylene chain offers the largest number of internal degrees of freedom of the three, and its response is distributed over the chain rather than concentrated at the interface.

HMDI-PANI reverses the pattern and amplifies it. R_1_ shortens by 0.00089 Å and R_2_ lengthens by 0.00122 Å, so the left bond contracts while the right bond expands. The combined modulation reaches 0.00211 Å, nearly five times the 0.00045 Å of HDI-PANI. The dicyclohexyl scaffold has the fewest internal degrees of freedom of the three, and the field-induced change of the electron density is expressed at its junction bonds instead of being distributed over the chain.

IPDI-PANI falls between the two extremes in magnitude but follows the same sign pattern as HDI. R_1_ lengthens by 0.00072 Å. R_2_ shortens by 0.00071 Å. Its R_2_ bond is notably longer at zero field, at 1.39406 Å compared to around 1.383 Å for the other two systems, which suggests weaker initial bonding at the right PANI interface. The isophorone ring introduces steric congestion near the graft junction that weakens the right-side C-N bond relative to the left.

The bond length data establish two points. HMDI-PANI carries the largest junction change of the three, and HDI-PANI the smallest. The sign pattern also differs: HDI-PANI and IPDI-PANI accumulate charge at the right interface, and HMDI-PANI at the left.

The changes reported here are small on the scale of the vibrational amplitude of the same bond. Summing the normal modes of the optimized structure gives a zero-point amplitude of 0.035 to 0.057 Å for the two junction bonds, which is 159 to 577 times the changes listed in Table 1, so the junction bond is not read as displaced by the field. What the change reports is the electron population of the bonding region, and its role in the argument is the ordering it produces among the three systems under an identical protocol. Within each system the two junctions move in opposite directions, one contracting while the other expands, which is the signature of a directional displacement of charge along the field axis.

As shown in Figure 2b,c, IPDI-PANI exhibits the largest polarizability increase, at 9.0 a.u., and the largest dipole moment enhancement, at 1.29 D. The isophorone ring carries two pendant methyl groups attached to quaternary and tertiary carbons. These methyl substituents are not locked into a rigid ring structure. They reorient under the external field, creating additional induced dipoles. IPDI also has the largest zero-field polarizability among the three at 1161.6 a.u., a highly polarizable baseline that the field can push further.

HDI-PANI is the one system in which the two quantities move in opposite directions. Its polarizability rises monotonically by 4.04 a.u., from 1110 to 1114 a.u., while its dipole moment falls by 0.24 D, from 7.54 to 7.29 D, and passes through a minimum of 7.165 D at 102.8 kV/mm. The two quantities are different in kind. The polarizability is the response coefficient of the electron cloud to the applied field, whereas the dipole moment is a property of the structure obtained by geometry optimization at that field strength, so its field dependence follows the change of that structure as well as the electronic response. A rising polarizability and a falling dipole magnitude can therefore appear in the same system, and the text states this distinction instead of implying that the two must move together.

HMDI-PANI sits between the two extremes with minimal changes, a Δα of 1.1 a.u. and a Δ*μ* of 0.23 D. The dicyclohexyl scaffold resists both conformational deformation and field-induced electronic polarization. It is mechanically rigid and electronically stiff.

The contrast between the bond-length and polarizability data is instructive. HMDI shows the largest interfacial bond response but only a modest polarizability change. IPDI shows the largest polarizability increase but only a moderate bond response. The two metrics measure different things. Bond strain is local. It reports the electron density at a single covalent contact linking PANI to PU. Polarizability is global. It integrates every dipole induced anywhere in the molecule, whether that dipole contributes to interfacial charge transfer or not.

This difference traces back to the hard segment architecture. The isophorone ring in IPDI carries methyl substituents that are free to reorient under the field. These methyl groups contribute strongly to the polarizability increase but their motion is spatially remote from the graft junction. They create local dipoles on the PU side that do not translate into mechanical strain at the C-N bonds. The polarizability rises, but the graft interface barely feels it.

The dicyclohexyl scaffold in HMDI works in the opposite way. Each cyclohexyl ring is directly attached to the urethane linkage that forms the graft junction. The rings are rigid. They do not deform internally. When the field applies stress, the entire scaffold moves as a unit and transmits that stress to the interfacial C-N bond. The polarizability change is small because the scaffold resists internal polarization. The bond strain is large because the scaffold transfers mechanical force efficiently from the PU donor region to the PANI acceptor.

HDI-PANI follows a third route. Its hexamethylene chain offers the largest number of internal degrees of freedom, and its junction bonds carry the smallest change of the three. The charge displaced by the field spreads over the flexible backbone instead of concentrating at the junctions, and the dipole magnitude is reduced even though the polarizability increases. The flexible chain separates the field response from the interface.

The three architectures partition the field response into distinct channels. HMDI transmits the field through its rigid scaffold directly to the interfacial bonds, producing large bond strain with little polarization. IPDI does the reverse. Methyl groups remote from the graft junction reorient freely and dominate the polarizability increase, while the interfacial bonds move only modestly. HDI avoids both outcomes. The flexible hexamethylene chain reorganizes conformationally and absorbs the stress before it reaches either the interface or the polarizable groups.

### 2.2. Bader Charge and Differential Charge Density Analysis

**Figure 3:**
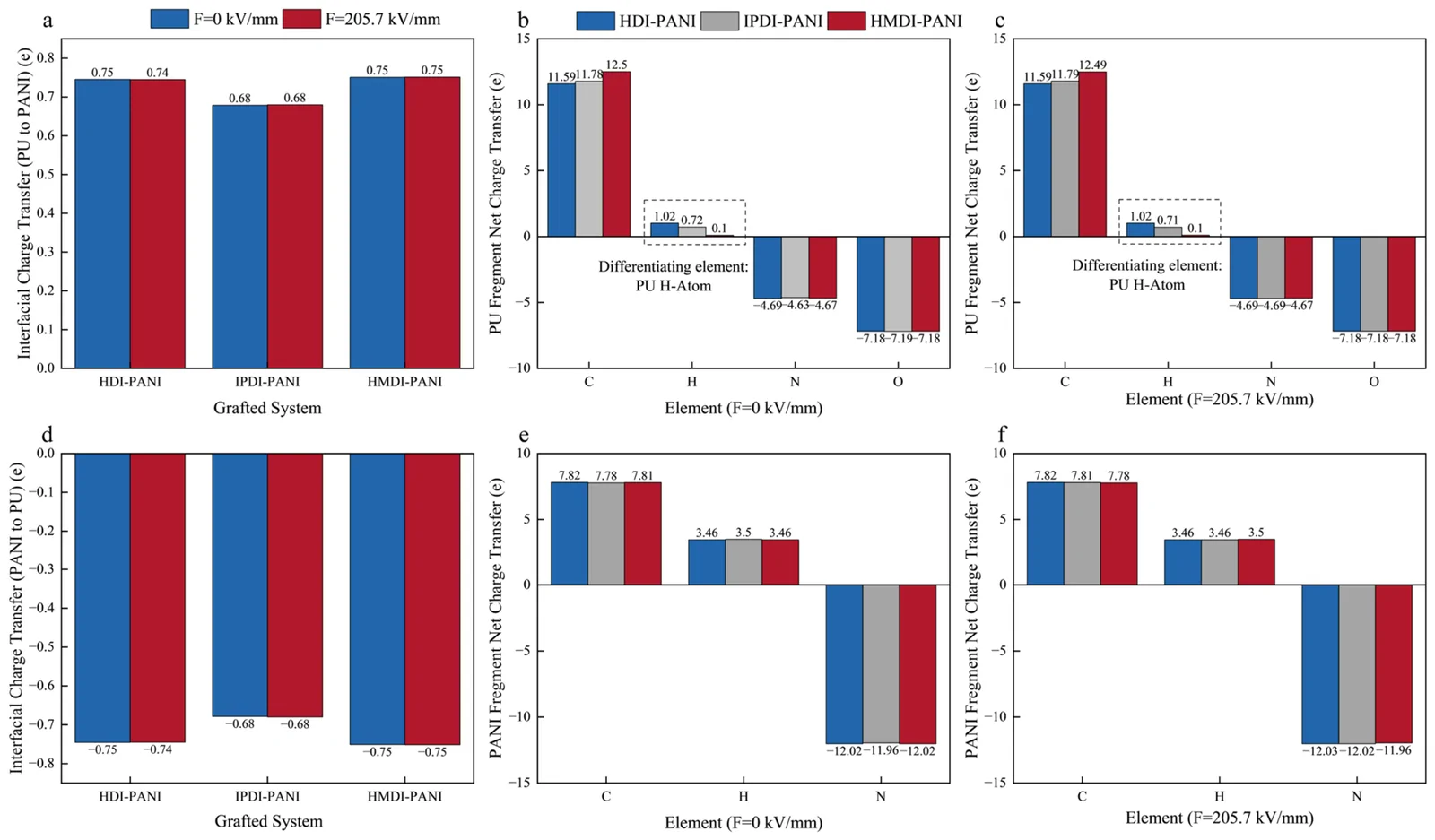
Bader charge analysis for PU (upper) and PANI (lower) fragments at F = 0 and F = 205.7 kV/mm. (**a**) Net charge transferred from PU to PANI (positive = electron loss from PU); (**b**,**c**) element-resolved charges of PU at F = 0 and F = 205.7 kV/mm; (**d**) Net charge accumulated on PANI (negative = electron gain); (**e**,**f**) element-resolved charges of PANI at F = 0 and F = 205.7 kV/mm. All values in elementary charge (**e**).

Figure 3 presents the Bader charge decomposition for all three systems, partitioned into PU and PANI fragments at zero field and at 205.7 kV/mm. The field changes almost nothing. The net charge transferred from PU to PANI is 0.74 e for HDI-PANI, 0.75 e for HMDI-PANI, and 0.68 e for IPDI-PANI, identical at both field strengths. Bader basin populations report the intrinsic polarity of the covalent bonds. A field of this magnitude shifts electron density within basins but does not push charge across basin boundaries.

Figure 3b,c break down the PU fragment by element. The heavy-atom contributions are nearly constant across the three hard segments. Oxygen gains 7.18 to 7.19 e. Nitrogen gains 4.63 to 4.69 e. All oxygen resides in the soft segment, which is identical across the three systems. The soft segment supplies the same amount of charge regardless of which diisocyanate it connects to. Carbon varies slightly more. HMDI carbon donates 12.50 e, compared to 11.59 e for HDI and 11.78 e for IPDI, reflecting the higher carbon density of the bicyclic scaffold.

Hydrogen diverges sharply. On the PU side, hydrogen loses 1.01 e in HDI, 0.72 e in IPDI, and only 0.10 e in HMDI, a tenfold difference. On the PANI side, hydrogen loses 3.73 e in HDI, 3.50 e in IPDI, and 3.46 e in HMDI. The PANI data mirror the PU data. Carbon and nitrogen on PANI are nearly constant. Hydrogen follows the hard segment ranking identically, though the spread is smaller because the PANI aromatic backbone has fewer C-H bonds and resists polarization.

The hydrogen variation traces to electronegativity. Hydrogen at 2.20 is less electronegative than carbon at 2.55. A polarized C-H bond pushes electron density from H toward C, leaving hydrogen with a positive Bader charge. The degree of polarization depends on whether the C-H bond is free to orient. HDI has twelve methylene C-H bonds that rotate freely and align with the field, summing to 1.01 e. HMDI has twenty C-H bonds locked into axial and equatorial orientations by the cyclohexyl rings, yet contributes only 0.10 e because fixed bonds cannot reorient. IPDI splits the difference. Six ring C-H bonds are locked, six methyl C-H bonds are free, and the total sits at 0.72 e.

This matters for charge transport. Each polarized C-H bond is a localized electrostatic dipole. A charge carrier moving from PU to PANI encounters these dipoles and is deflected. The cumulative effect is scattering that reduces the net flux reaching the acceptor. HDI activates twelve scattering centers. IPDI activates six. HMDI activates essentially none.

The carbon scaffold in HMDI compensates. HMDI carbon donates 12.50 e, the largest among the three, while hydrogen contributes almost nothing. Charge flows through carbon with minimal hydrogen-mediated scattering. HDI carbon donates only 11.59 e while hydrogen loses 1.01 e. More of the displaced charge is trapped in hydrogen dipoles than moves directionally through carbon. IPDI sits in the middle on both counts. The net charge transfer follows the same order. HMDI transfers 0.75 e, HDI transfers 0.74 e, IPDI transfers 0.68 e.

Where the Bader charges describe which atoms gain and lose charge, they cannot show where field-induced charge moves in space. The three-dimensional differential charge density addresses that question. Table 2 lists the volumetric metrics. The total redistribution, obtained by integrating |Δρ| over the full grid, ranks HMDI first at 0.0793 e, followed by IPDI at 0.0765 e and HDI at 0.0628 e. This order differs from the Bader transfer order. The comparison of the two rankings is diagnostic. Bader counts charge that crosses basin boundaries. The DCD counts all charge that moves, including charge that shifts within a single basin without going anywhere. IPDI has the second-largest DCD redistribution but the smallest Bader transfer of 0.68 e. Much of the charge set in motion stays inside the PU fragment. The isophorone ring polarizes locally and the methyl groups reorient, but neither event pushes charge across the graft junction. HMDI, by contrast, combines the largest redistribution with the largest Bader transfer of 0.75 e. Charge that moves in HMDI reaches the acceptor. The field-induced motion couples efficiently to net transfer.

The diffuseness index, defined as the ratio of total redistribution to peak density, captures how the moving charge is spread. HMDI achieves 122.6, against 75.2 for HDI and 80.8 for IPDI. HMDI distributes its charge over 2286 Bohr^3^, the largest volume, with the lowest peak density of 0.647 × 10^−3^ e/Bohr^3^. IPDI shows the opposite character. Its peak density of 0.947 × 10^−3^ e/Bohr^3^ is the highest, indicating that charge piles up at specific sites rather than spreading out. The spatial entropy, which measures how many distinct features the charge distribution contains, is highest for IPDI at 11.39. The isophorone ring, the soft segment center, and both PANI termini all participate in IPDI. HDI has the lowest entropy at 10.93, consistent with injection concentrated at a single PANI terminal.

**Figure 4:**
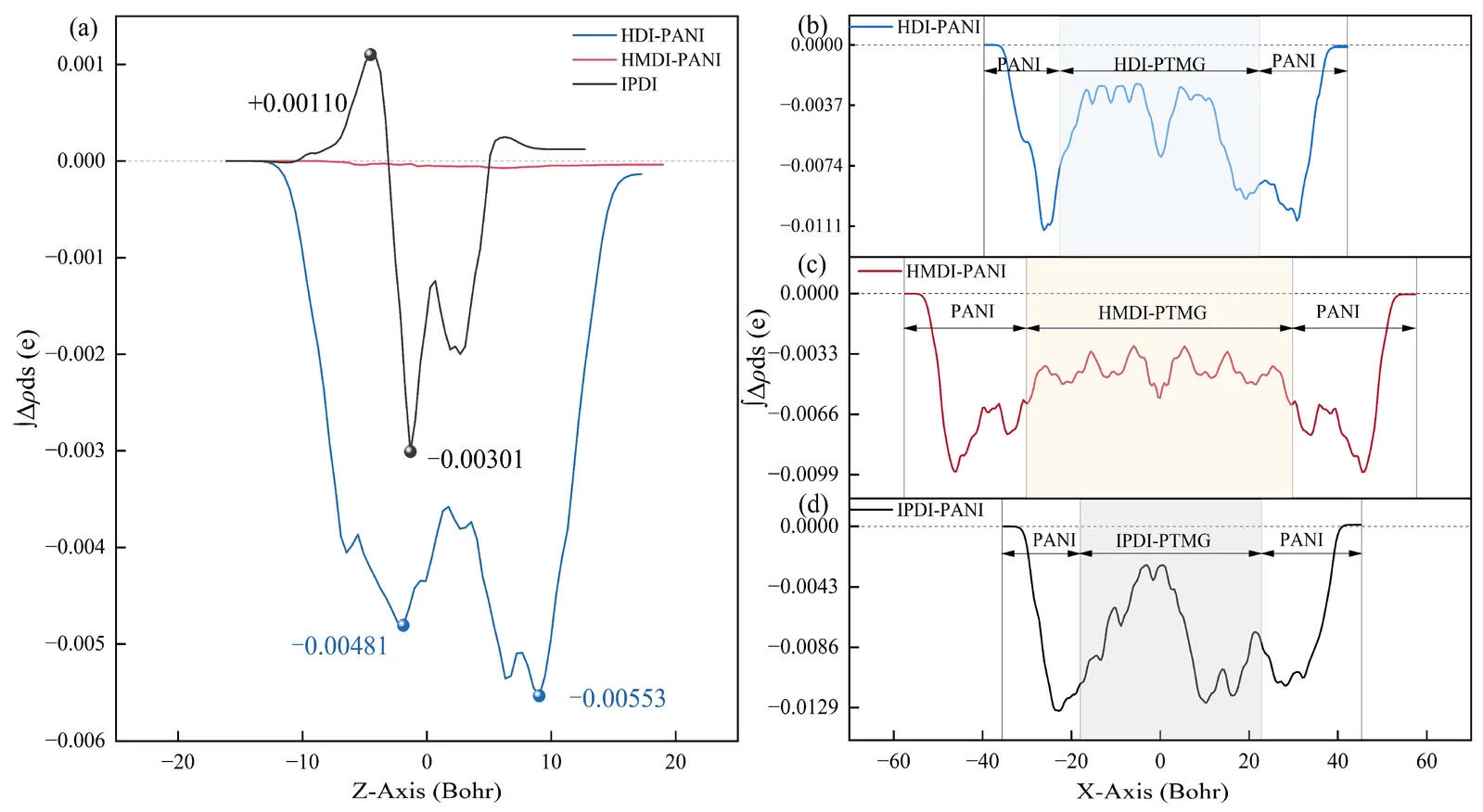
Plane-integrated electron density difference Δ*ρ* = *ρ*(205.7 kV/mm) − *ρ*(0). (**a**) Z-axis projection; (**b**–**d**) X-axis projections for HDI-PANI, HMDI-PANI, and IPDI-PANI.

The one-dimensional integral curves place these volumetric patterns along the molecular axes. In the Z-axis projection of Figure 4a, which integrates Δ*ρ* over the XY plane at each height, HDI-PANI gives the most structured profile of the three, with a peak-to-valley amplitude of 0.00554 and its two deepest minima at Z ≈ +6.2 and +9.1 Bohr. IPDI-PANI oscillates at about 0.004, mixing junction-localized and soft-segment contributions. HMDI-PANI is almost featureless. Its amplitude is roughly two orders of magnitude smaller, so the laterally moving charge never accumulates at any particular height, and this flatness is the direct counterpart of the diffuseness index reported above.

The X-axis projections in Figure 4b–d resolve the redistribution along the field direction, with the PANI–Hard–Soft–Hard–PANI architecture overlaid. All three curves fall sharply at the PANI boundaries and level off inside the chains. HDI-PANI reaches −0.0114, its two deepest valleys lying at X ≈ −26.3 and +30.8 Bohr; the redistribution is therefore bilateral, the left PANI region being slightly deeper than the right, while the central soft-segment region stays shallower. HMDI-PANI gives the shallowest integral of the three at −0.0099, with a flat PANI centre, so that charge crosses the interfaces and spreads along the conjugated chains without accumulating. The deepest integral belongs to IPDI-PANI, whose central soft-segment region drops further than in the other two systems, indicating a contribution from the polyol chain as an additional electron source.

The hydrogen Bader charge ties the atomic and spatial pictures together. HDI carries 1.02 e of hydrogen polarization and oscillates at 0.00554 in the Z projection. IPDI carries 0.72 e and oscillates at about 0.004. HMDI carries 0.10 e and is nearly flat. The relationship is monotonic. More C-H polarization produces more spatial charge localization. Less C-H polarization produces a more diffuse charge distribution. The hydrogen neutrality that distinguishes HMDI is the common origin of its flat Z-axis profile, its flat PANI-center X-axis profile, its high diffuseness index, and its efficient coupling of field-induced motion to net charge transfer.

### 2.3. Electronic Structure and Frontier Orbital Response

**Figure 5:**
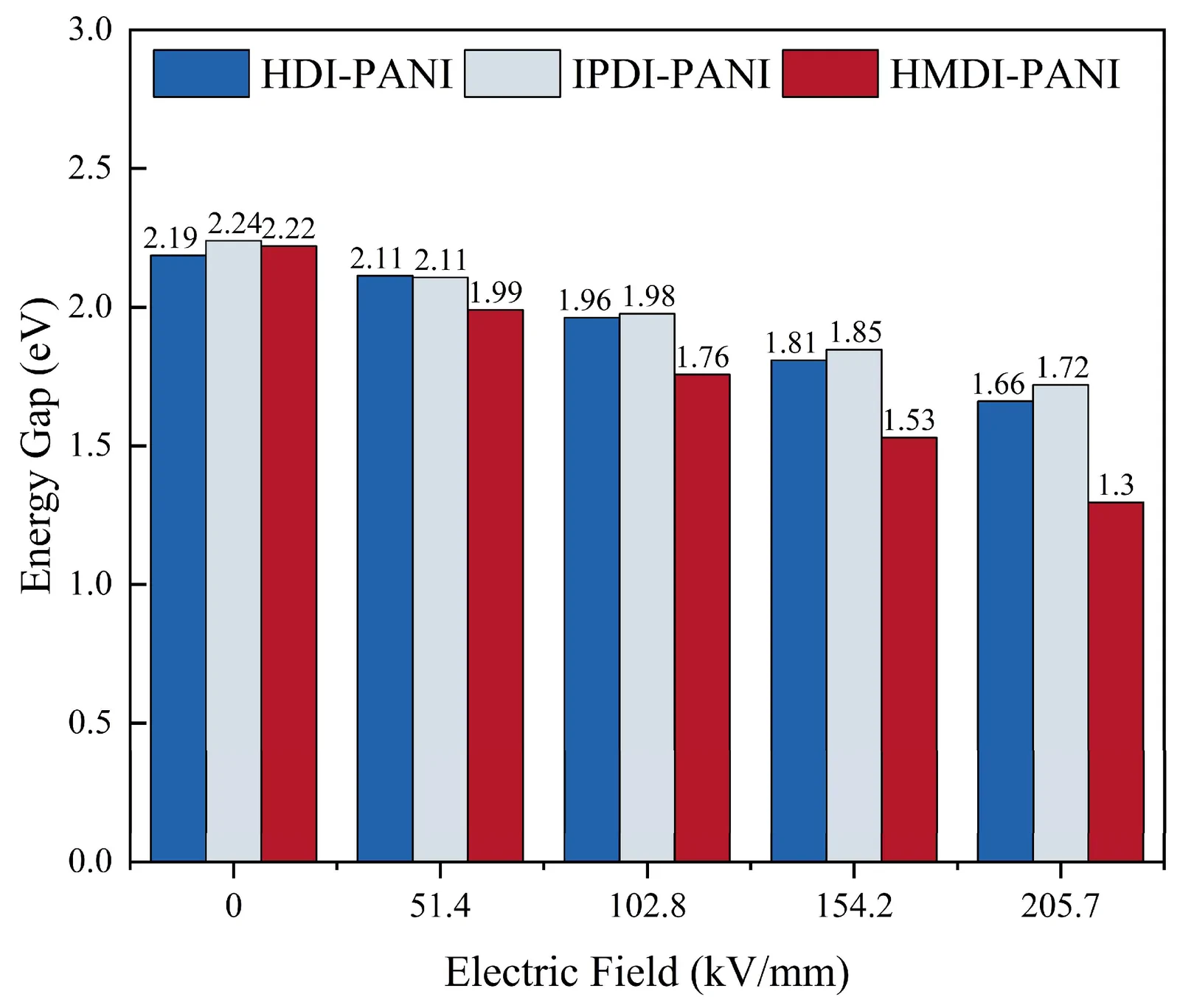
Comparison of the energy gap (Eg) for HDI-PANI, HMDI-PANI, and IPDI-PANI under applied external electric fields ranging from 0 to 205.7 kV/mm.

**Figure 6:**
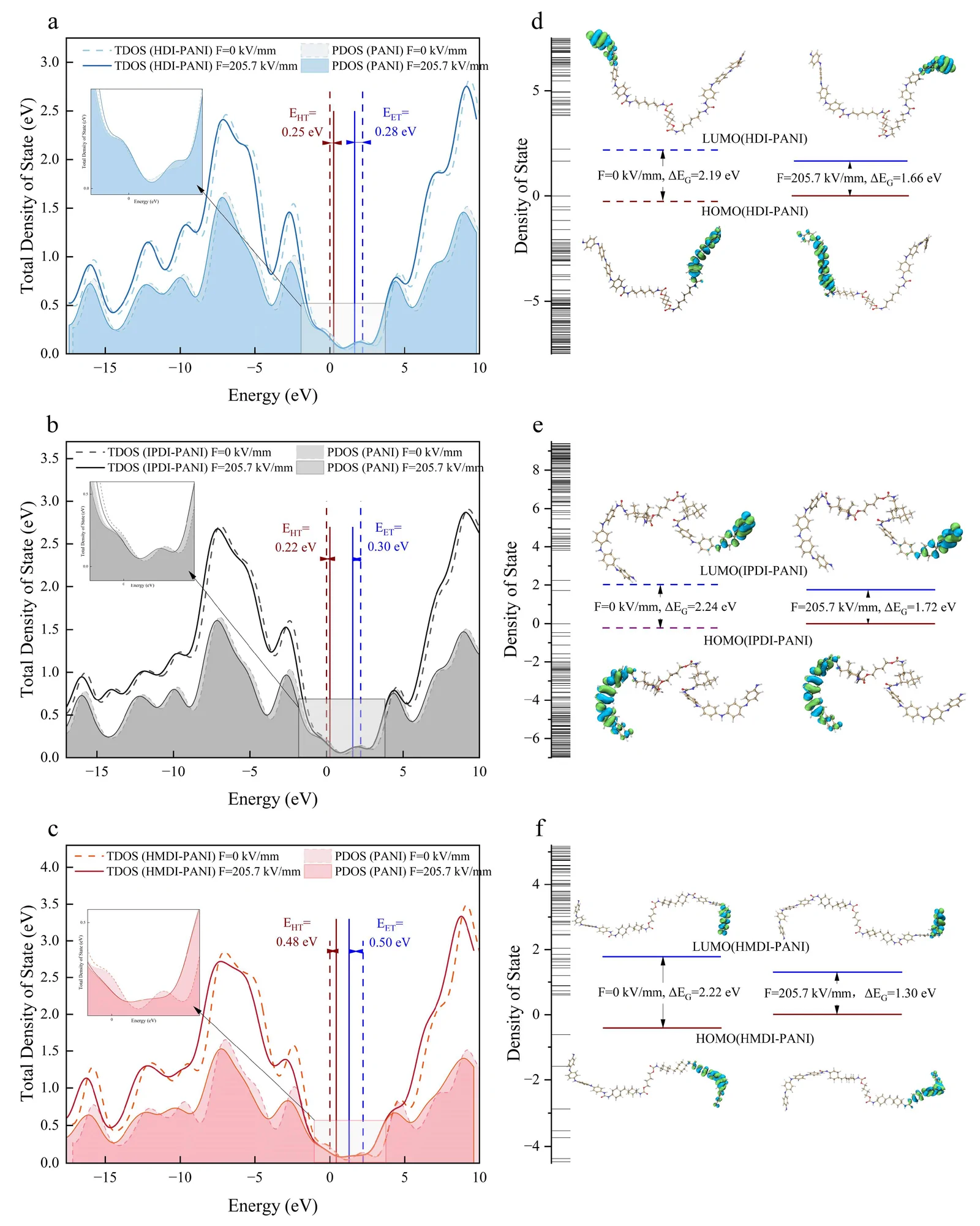
Density of states (DOS) and frontier molecular orbital (FMO) analysis at zero field (dashed lines) and at the maximum applied field of 0 to 205.7 kV/mm (solid lines). (**a**–**c**) Total DOS curves for HDI-PANI, IPDI-PANI, and HMDI-PANI; the filled areas represent the projected DOS (PDOS) contributions from the PANI fragments. (**d**–**f**) HOMO–LUMO energy level diagrams for the three systems at both field conditions. The dashed line represents the orbit at zero field, while the solid line represents the orbit at maximum electric field.

Figure 5 tracks the energy gap as a function of the applied field. Figure 6 presents the DOS curves and frontier orbital distributions at zero field and at 205.7 kV/mm.

At zero field the three systems have comparable gaps. HDI sits at 2.19 eV, IPDI at 2.24 eV, and HMDI at 2.22 eV. The differences are small, consistent with the similar geometries of the three copolymers. The field response separates them.

HDI-PANI has an energy gap of 2.19 eV, and the gap narrows steadily as the field rises. It falls to 2.11 eV at 51.4 kV/mm, 1.96 eV at 102.8 kV/mm, 1.81 eV at 154.3 kV/mm and 1.66 eV at 205.7 kV/mm, a net decrease of 0.53 eV over the range studied. The narrowing is driven from both sides of the gap. The HOMO rises by 0.25 eV while the LUMO drops by 0.28 eV, and these two shifts together account for the entire gap change. Closing of the gap under an external field is the expected behaviour, because the field mixes occupied and virtual orbitals and pushes their energies apart. The response is gentler in the first interval, about 0.0014 eV per kV/mm against 0.0029 eV per kV/mm afterwards, and no plateau appears up to 205.7 kV/mm.

All three systems narrow with the field, but at different rates. IPDI decreases from 2.24 to 1.72 eV, a reduction of 0.52 eV. HMDI decreases from 2.22 to 1.30 eV, a reduction of 0.92 eV. The dicyclohexyl scaffold shows the smallest geometric response of the three, so the field acts on the electronic degrees of freedom and the gap closes sharply. At 1.30 eV, HMDI approaches the narrow-bandgap regime.

Figure 6a–c overlay the TDOS and PANI-projected PDOS at both field conditions. The dashed lines mark zero field, the solid lines mark the maximum field, and the shaded areas show the PANI contribution to the density of states. In all three systems the field shifts the DOS toward lower energy. PANI orbitals dominate the band-edge states regardless of hard segment. The hard segment affects the energy position and width of these states, not their PANI versus PU character.

The hole injection barrier E_HH_ and electron transfer barrier E_ET_, marked on the same panels, provide additional differentiation. HDI has the smallest E_ET_ at 0.28 eV. The HOMO sits close to the zero energy point. HMDI has the largest barriers at 0.48 and 0.50 eV. IPDI falls in between at 0.22 and 0.30 eV. Large local barriers mean that injecting a single carrier requires more energy. But the total charge transmitted through the interface integrates over all available transport channels. HMDI compensates for higher barriers with a greater number of delocalized states near the band edges.

Figure 6d–f show the HOMO and LUMO isosurfaces. In HDI-PANI the field drives the two frontier levels toward each other, the HOMO rising from −4.60 to −4.35 eV and the LUMO falling from −2.42 to −2.69 eV, so the gap narrows from 2.19 to 1.66 eV. The right PANI chain contributes little to the highest occupied state. In HMDI, The gap closes most in HMDI-PANI, from 2.22 to 1.30 eV, with the HOMO climbing from −4.60 to −4.18 eV and the LUMO dropping from −2.38 to −2.88 eV. The HOMO is distributed along the same PANI chain in both cases, indicating a relatively low degree of response to the electric field. IPDI-PANI keeps the widest gap of the three at every field, closing from 2.24 to 1.72 eV, as its LUMO falls from −2.51 to −2.81 eV and its HOMO rises less, from −4.75 to −4.53 eV. The HOMO localizes on one PANI chain at zero field, similar to HDI, but under the field it shifts to involve the soft segment region. The isophorone ring and its methyl substituents begin to contribute to the highest occupied state.

The HOMO keeps the same spatial character at zero field and at the maximum field and stays on the same PANI chain throughout the scan, so the field shifts the frontier level positions of this system without relocating the highest occupied state.

### 2.4. Electrostatic Potential and Electron Localization Function Analyses

**Figure 7:**
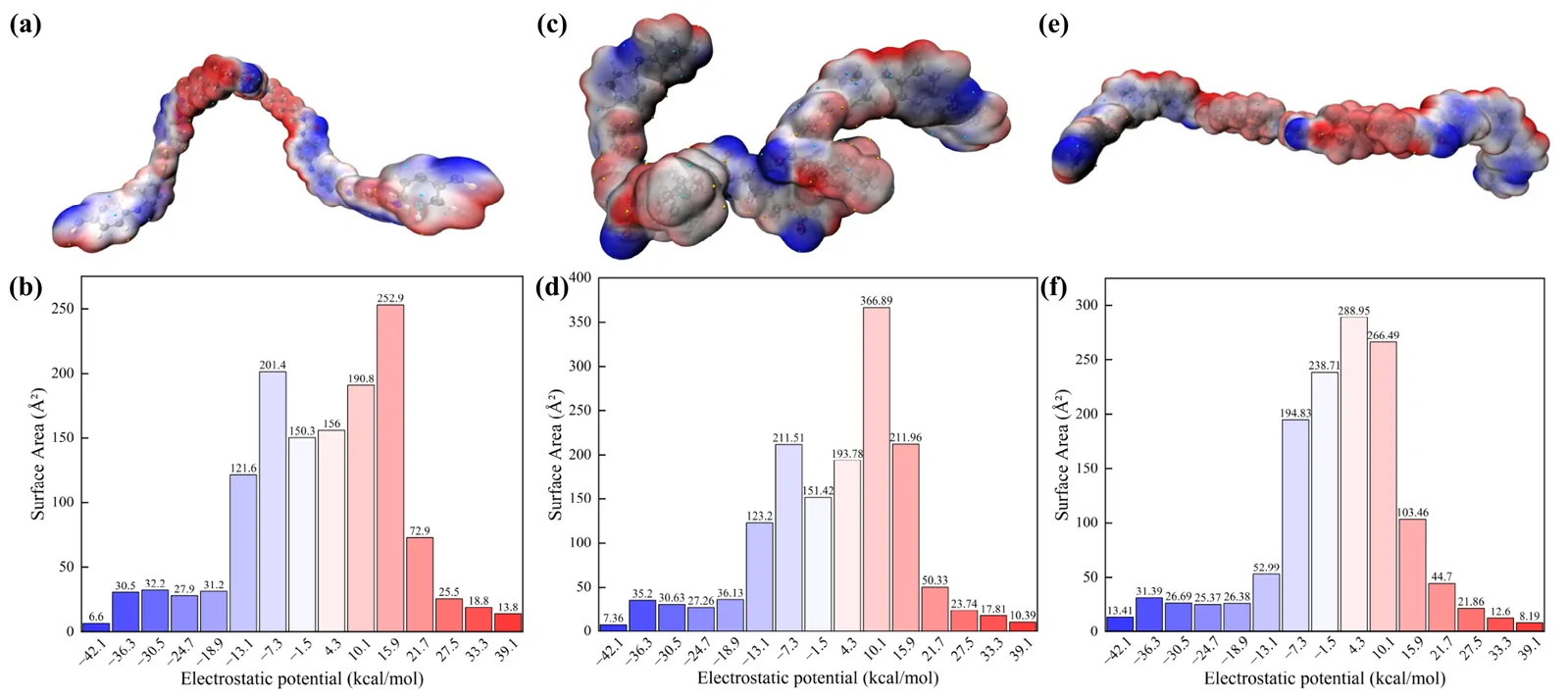
Electrostatic potential (ESP) mapped onto the molecular Van der Waals surface for (**a**) HDI-PANI, (**c**) IPDI-PANI, and (**e**) HMDI-PANI, with the corresponding surface-area histograms shown in (**b**,**d**,**f**).

HDI-PANI lies as a wavy chain whose two PANI arms point away from each other, shown in panels a and b of Figure 7. Blue regions of electron accumulation cover the terminal rings and the top of the central bridge, while red regions of electron depletion run along the middle of the chain. The area histogram peaks at +15.9 kcal/mol with 252.9 Å^2^, and the distribution spans −42.1 to +39.1 kcal/mol, the widest range of the three. Positive potential covers 54.8% of the surface, so charge separation is present but limited.

IPDI-PANI has the most concentrated pattern of the three, with electron-rich and electron-poor regions close together on a compact body, shown in panels c and d of Figure 7. Blue lobes cover the outer ends of the arms and red patches lie in the gaps between them. Its histogram peaks lowest at +4.3 kcal/mol with 289.0 Å^2^, and the window within plus or minus 5.8 kcal/mol holds 794.2 Å^2^, about 2.4 times the HDI-PANI value. The weighted mean potential is +1.31 kcal/mol, the closest to neutral of the three.

HMDI-PANI shows a stronger positive shift. Its histogram maximum is the largest of the three at 366.9 Å^2^, and it sits at +10.1 kcal/mol, as shown in panels e and f of Figure 7. Blue and red patches alternate along the whole chain, so terminal and central domains do not stand out on this surface. Positive potential covers 58.4% of the surface, the largest share of the three, while the weighted mean potential stays near neutral at +2.26 kcal/mol.

**Figure 8:**
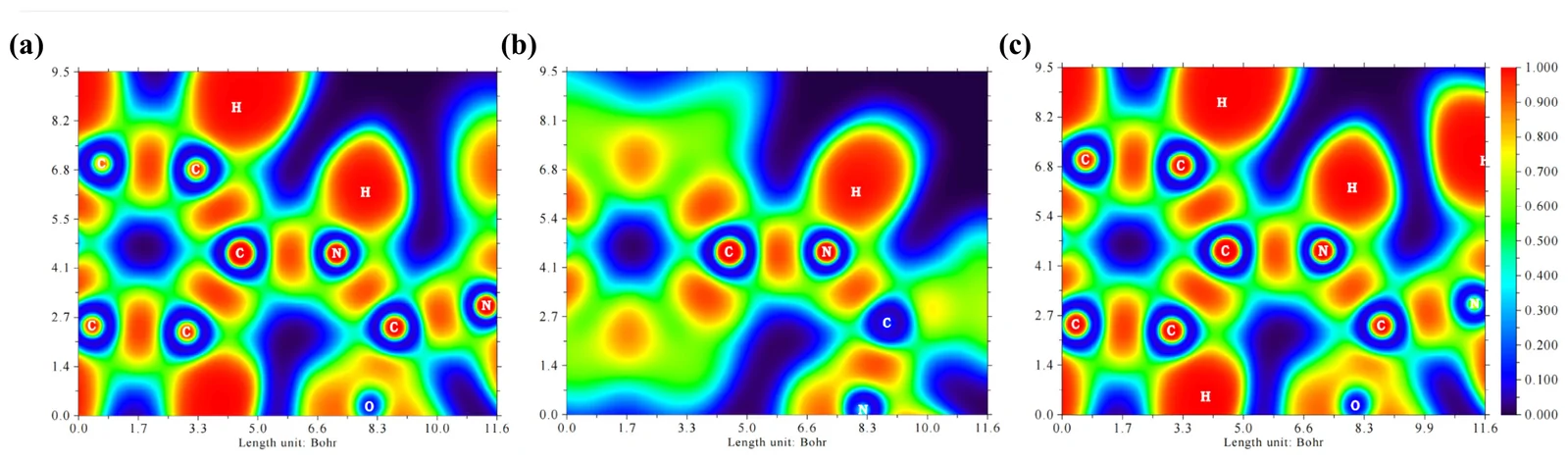
Electron Localization Function (ELF) contour maps at the graft junctions (C–N bonds connecting the diisocyanate hard segment to the PANI terminal NH groups) for (**a**) HDI-PANI, (**b**) IPDI-PANI, and (**c**) HMDI-PANI under 205.7 kV/mm. The color scale ranges from 0 (blue, low electron localization) to 1 (red, high electron localization).

Figure 8 presents ELF contour slices through the graft junction plane. The ELF scale runs from 0, corresponding to a uniform electron gas, to 1, corresponding to perfect localization in a covalent bond or lone pair. In all three systems the PANI aromatic rings show intermediate ELF values between rings, consistent with the partial π-delocalization of the emeraldine salt state. The absolute inter-ring ELF values differ among the three systems, with HMDI showing the lowest values, indicating the greatest degree of delocalization within the PANI backbone.

At the C-N graft junction the ELF registers values of 0.5 to 0.6, characteristic of a polar covalent single bond. The electron density at the junction is localized between the carbon and nitrogen nuclei. The graft junction is a discrete bonding contact, not an extension of the PANI conjugation. The degree of localization varies with the hard segment. A more localized junction, with ELF values nearer 0.6, presents a sharper potential step for charge crossing from PU to PANI. A less localized junction, with values shifted slightly downward, indicates a more gradual electronic transition.

The hard segment bonding patterns match the expected chemistry. HDI shows a regular array of isolated C-C and C-H bonding basins with ELF near 0.8 to 0.9 along the hexamethylene chain. Each methylene group is electronically isolated. HMDI shows geometrically constrained C-C bonds within the cyclohexyl rings with marginally reduced ELF at ring junctions, reflecting ring strain that partially delocalizes the σ-framework. IPDI shows asymmetric ELF distribution around the quaternary carbon of the isophorone ring, consistent with the steric inequivalence of its substituents. The C-H bonds on the methyl groups are the most localized features in any of the three systems.

The ESP and ELF data converge on the same three mechanisms. HDI segregates charge spatially and localizes each bond into discrete σ-basins. Charge transfer must cross a series of isolated steps from soft segment through hard segment to the graft junction. HMDI spreads charge evenly on the surface and its ring-strained C-C bonds show marginally reduced localization. The more gradual electronic transition facilitates diffuse transfer. IPDI produces the most polarized surface and the most asymmetric bond localization, particularly at the quaternary carbon. The spatial complexity underlies the mixed polarization mechanism in which methyl-group reorientation competes with directional charge transfer.

## 3. Discussion

The diisocyanate hard segment does not merely shift the magnitude of the charge transferred between PU and PANI; it selects the mechanism by which the field-induced charge moves. Within the isolated-chain, static-field model adopted in this work, the independent analyses of geometry, charge partitioning, differential density, orbital energetics and surface potential converge on the same picture, and the hard segment determines which channel of response dominates.

The HMDI-based system operates by rigid coupling. Its dicyclohexylmethane scaffold shows little structural deformation under the field, and its twenty C–H bonds, locked in fixed orientations, contribute only 0.10 e of parasitic hydrogen polarization, the smallest contribution of the three systems. The field stress, denied a mechanical outlet, is transmitted to the interfacial C-N bonds, and the same rigidity lets the energy gap narrow by 0.92 eV. This coupling explains why HMDI tops every independent ranking in the study. It combines the largest Bader carbon donation of 12.50 e with the smallest hydrogen polarization, the highest diffuseness index of 122.6, the flattest Z-axis profile, and the largest net charge transfer of 0.75 e. The result is a directional and spatially uniform response in which the field acts on the electronic degrees of freedom rather than on the molecular skeleton.

The HDI-based system operates by flexible-chain accommodation. The hexamethylene chain gives the smallest geometric response of the three, and the field response appears in the polarization rather than at the junctions. The polarizability rises by 4.0 a.u. while the dipole moment falls by 0.25 D and passes through a minimum at 102.8 kV/mm, and the total junction-bond modulation, 0.00045 Å, is the smallest of the three systems. The induced polarization is thus redistributed along the flexible backbone instead of appearing as a net dipole. The gap narrows by 0.53 eV, second only to HMDI, and the plane-integrated density difference is deep at both PANI regions, so that the field-induced redistribution is bilateral rather than confined to a single terminal. The C–H bonds that polarize under the field carry a charge of 0.75 e and then provide scattering sites for the charge that crosses the interface.

The IPDI-based system follows a mixed polarization mechanism. Six constrained ring C-H bonds and six free methyl C-H bonds yield an intermediate hydrogen charge of 0.72 e. The methyl groups drive the largest polarizability increase, at 9.0 a.u., and the largest dipole enhancement, at 1.29 D, but this polarization does not translate into net interfacial transfer. The Bader net transfer of 0.68 e is the smallest, because the methyl groups act remote from the graft junction. They polarize locally without pushing charge across the C-N bonds. The activated soft segment appears as the deepest X-axis integral and the soft-segment-weighted HOMO. Consistently, the electrostatic potential of IPDI-PANI is the most uniformly polarized and overall the most neutral surface of the three systems.

The hydrogen Bader charge on the PU fragment is the single most useful predictor to emerge from this comparison. Across all three architectures, it correlates with every independent measure of transfer quality. Near-zero hydrogen charge, as in HMDI, routes the field response through the carbon framework with minimal scattering. Large hydrogen charge, as in HDI, dissipates field energy into C-H dipole formation. Polarizability is not a reliable screening metric for interfacial transfer. IPDI has the largest Δ*α* and the largest Δ*μ*, yet the lowest net charge transfer. A screening strategy that ranked hard segments by polarizability alone would select the worst performer.

Read together with the bond-strain data, the hydrogen-charge descriptor suggests the structural conditions for efficient transfer. A hard segment should respond to the field mainly through its electronic rather than its geometric degrees of freedom, supply charge from a carbon-rich framework, and avoid freely rotating substituents that generate parasitic polarization. The dicyclohexylmethane scaffold meets these conditions within the present model, which rationalizes why it outperforms both the flexible linear chain of HDI-PANI and the methyl-bearing cycloaliphatic ring of IPDI-PANI among the segments tested here.

The present results are based on single copolymer chains in vacuum at a static field of 205.7 kV/mm, so they should be interpreted with the usual care. A realistic coating contains entangled networks, amorphous domains, and residual moisture that will modulate the field response. Lightning-induced fields are dynamic and can reorient on microsecond timescales. Only one oxidation state of PANI, the emeraldine base, was treated. The hydrogen neutrality principle is directly testable. If the rigid, carbon-rich scaffold behaves as predicted, the HMDI variant should show the lowest volume resistivity under field among the three copolymers.

## 4. Materials and Methods

### 4.1. Molecular Models

**Figure 9:**
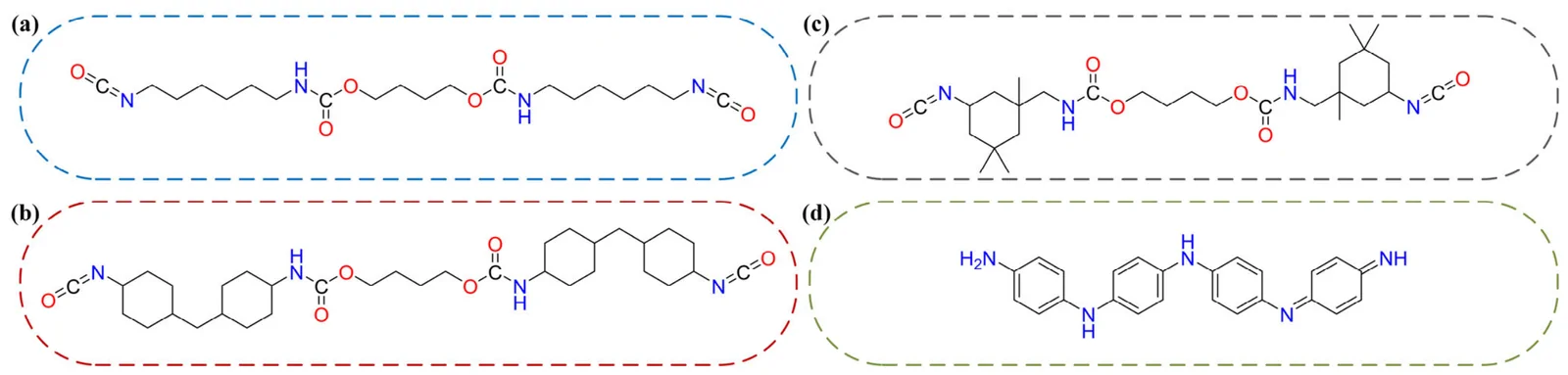
Molecular structures of the building blocks of the polyaniline-grafted polyurethane copolymers. (**a**) The HDI-PTMG polyurethane segment, (**b**) the IPDI-PTMG polyurethane segment, (**c**) the HMDI-PTMG polyurethane segment, and (**d**) the polyaniline chain in its emeraldine base (EB) oxidation state.

Three PANI-grafted polyurethane copolymers were constructed to represent the graft interface between the conductive polyaniline component and the polyurethane matrix. The materials used are the polyurethane segments HDI-PTMG, IPDI-PTMG, and HMDI-PTMG, each composed of the corresponding diisocyanate hard segment and the central PTMG soft segment, and the polyaniline chain in its Emeraldine Base (EB) oxidation state with eight aniline repeat units [29,30], as illustrated in Figure 9. Each model comprises two PANI chains covalently linked at both termini to the PU segment through C–N bonds, forming a symmetric five-block architecture of PANI–Hard Segment–Soft Segment–Hard Segment–PANI [24]. The resulting copolymers contain about 150 to 190 atoms, providing an extended π-conjugated backbone while remaining computationally tractable [31,32].

### 4.2. Computational Details

All DFT calculations were performed with Gaussian 16. Geometry optimizations and frequency calculations were carried out at the B3LYP/6-31G(d,p) level of theory, a combination well established for organic polymers and donor–acceptor systems [33,34,35,36,37].

A static external electric field was applied along the +X molecular axis during both optimization and frequency steps, with field strengths ramped from 0 to 4 × 10^−4^ a.u. (approximately 205.7 kV/mm). The +X direction points from the left PANI chain toward the right PANI chain along the molecular backbone.

All systems were optimized and characterized under an identical protocol, so the comparisons drawn in this work are internal to the set and do not rest on absolute magnitudes.

Polarizabilities were obtained as the isotropic mean of the polarizability tensor of the optimized structure at each field strength, and the dipole moments are the magnitudes of the corresponding SCF dipole vectors. The two quantities are therefore of different kinds, one a response coefficient and the other a property of the structure.

Frequency calculations were performed at every field strength, from zero to the maximum value of 205.7 kV/mm. Every optimized structure has zero imaginary frequencies, so the complete set of 3N − 6 vibrational modes is real and each reported structure is a genuine minimum on the potential energy surface rather than a saddle point.

This holds at the maximum field as well, where the conjugated backbone carries the strongest field-induced polarization. The applied field therefore modifies the electronic structure and the charge distribution without producing a structural instability of the backbone.

Wavefunction analysis and electron density topology were conducted using Multiwfn 3.8 [38,39]. The following analyses were carried out on the converged wavefunctions at zero field and at the maximum applied field:

(1). Bader charge partitioning was performed using the grid-based Bader algorithm implemented with a grid spacing of 0.05 Bohr, yielding basin charges converged to within 10^−6^ e [40,41].

(2). The three-dimensional differential charge density was computed through wavefunction analysis of the converged wavefunctions at F_0_ = 0 and F_max_ = 205.7 kV/mm, and exported as Gaussian cube files. The field-induced density difference Δ*ρ*(r) is defined in Equation (1). Plane-integrated projections along the molecular axes were obtained by integrating Δρ over the orthogonal two dimensions, yielding the X-axis and Z-axis profiles of Equations (2) and (3), respectively. The X-projection skewness and forward charge fraction were computed to characterize the asymmetry of the field-induced charge redistribution [42].

(1) Δρ(r)=ρ(E,r)−ρ(0,r)

(2) I(X)=∬Δρ(x,y,X)dxdy

(3) I(Z)=∬Δρ(x,y,Z)dxdy

(4) $$Q_{\mathrm{Total}}=\int \left|\Delta \rho \right|dV$$

(5) $$D=\frac{\int \left|\Delta \rho \right|dV}{\max(\left|\Delta \rho \right|)}$$

(6) $$\begin{array}{l} S=-\sum p_{i}\ln(p_{i})\\ p_{i}=\left|\frac{\Delta \rho (r_{i})}{\sum \Delta \rho (r_{i})}\right| \end{array}$$

(7) $$\mu_{\mathrm{DCD}}=\int r\cdot \Delta \rho (r)dV$$

(3). Total and projected densities of states (TDOS and PDOS) were computed from the molecular orbital energies at the optimized geometries. The PDOS resolved the orbital contributions from the PANI and PU components separately.

(4). The total charge redistribution was obtained by integrating |Δ*ρ*(r)| over the full grid (Equation (4)). The diffuseness index, defined as the ratio of total redistribution to the peak absolute density (Equation (5)), characterizes how the moving charge is spread. The spatial entropy was calculated according to Equation (6), summed over all grid points with |Δ*ρ*| > 10^−5^ e/Bohr^3^. The DCD-induced dipole moment was computed as the first moment of Δ*ρ*(r) (Equation (7)).

Throughout this work, rigidity denotes the resistance of a hard segment to field-induced structural reorganization. It is quantified by the response of the optimized geometry and of the electronic structure along the applied field, and it is therefore an electronic-structure-level property of the covalent scaffold, set by the curvature of the potential-energy surface in the direction of the perturbation. It is not to be confused with conformational relaxation dynamics, the thermally activated interconversion of conformers, which is not addressed in this study. Within this definition the evidence for the contrast between the segments is direct. Under a same basis set and functional, HMDI-PANI responds almost entirely electronically, HDI-PANI accommodates the field through restructuring of its flexible chain, and IPDI-PANI through local reorientation of its pendant methyl groups.

## 5. Conclusions

DFT calculations were employed to compare three PANI-grafted polyurethane copolymers that differ only in their diisocyanate hard segment under an external electric field. The HDI, HMDI, and IPDI architectures produce three distinct charge regulation mechanisms that diverge in the spatial distribution, energetics, and efficiency of field-induced charge transfer.

The HMDI-based system achieves the most efficient transfer through a scaffold that responds electronically rather than structurally. Its dicyclohexyl scaffold shows the smallest geometric response of the three, locks twenty C-H bonds into fixed orientations that contribute only 0.10 e of parasitic hydrogen polarization, and its junction bonds carry the largest change of the three. The energy gap narrows by 0.92 eV, the largest among the three, and charge spreads diffusely across both PANI chains. The flexible hexamethylene chain of HDI-PANI, by contrast, accommodates the field through structural reorganization. The IPDI-based system follows a mixed polarization mechanism in which freely rotating methyl groups drive the largest polarizability increase but do not translate this polarization into net charge transfer across the graft junction.

The hydrogen Bader charge on the PU fragment is a compact predictor of charge transfer efficiency. Across all three architectures, the PU hydrogen charge correlates with every independent measure of transfer quality. Near-zero hydrogen charge, as in HMDI, routes the field response through the carbon framework with minimal scattering. Large hydrogen charge, as in HDI, dissipates field energy into C-H dipole formation. Molecular polarizability, by contrast, is a misleading screening metric, since the system with the largest polarizability increase transfers the least charge.

Taken together, the results suggest that an efficient field-responsive hard segment should absorb the applied field mainly through its electronic rather than its geometric degrees of freedom, supply charge from a carbon-rich framework, and avoid freely rotating substituents that generate parasitic polarization. The dicyclohexylmethane scaffold meets these conditions within the present model, and the resulting prediction is directly testable on films of the three copolymers prepared under identical conditions, namely that the HMDI-based copolymer should show the lowest volume resistivity among the three under field.

## Figures and Tables

**Table 1:** C–N bond lengths at the PANI–PU graft junctions (Å). R_1_ and R_2_ are the individual C–N bonds at the two junctions, with R_a_ = (R_1_ + R_2_)/2. ΔR denotes the field-induced change at the maximum applied field.

System	R_1_ (F = 0)	R_1_ (F = Max)	ΔR_1_	R_2_ (F = 0)	R_2_ (F = Max)	ΔR_2_
HDI-PANI	1.38347	1.38356	0.00009	1.38327	1.38291	−0.00036
HMDI-PANI	1.38373	1.38284	−0.00089	1.38372	1.38494	0.00122
IPDI-PANI	1.38569	1.38641	0.00072	1.39406	1.39335	−0.00071

**Table 2:** Three-dimensional differential charge density (DCD) metrics for HDI-PANI, HMDI-PANI, and IPDI-PANI at F = 0 and F = 205.7 kV/mm.

Metric	HDI-PANI	HMDI-PANI	IPDI-PANI
Total redistribution (e)	0.0628	0.0793	0.0765
Peak |Δ*ρ*| (×10^−3^ e/Bohr^3^)	0.835	0.647	0.947
Redistribution volume (Bohr^3^)	1912	2286	2197
Diffuseness index	75.2	122.6	80.8
Spatial entropy	10.93	11.02	11.39

## Data Availability

The data that support the findings of this study are available from the corresponding author upon reasonable request.
